# Saving Exposed Pulps

**Published:** 1867-12

**Authors:** Chas. L. Houghton

**Affiliations:** Poughkeepsie, N. Y.


### SAVING EXPOSED PULPS

Is a theme upon which much has been, and more will be written by all classes of Dental writers, from which the practitioner often gains much valuable information, and quite as often the information gained is impracticable, worthless, and sometimes worse than worthless, as patients can testify who are victims of the experiments of Dentists who seize upon them to prove the correctness or incorrectness of the theory Dental writers frequently put forth as some new discovery or valuable information. The old aphorism, "Prove all things, hold fast that which is good;" is sound logic, and Dentists would be more successful and give better satisfaction to their patients, if they would take the old proverb as the text upon which to shape their practice; but proving whether certain theories and practices are correct or not at the peril of their patient's teeth and their own reputation, is a misdemeanor all honest Dentists should beware of.

I noticed in a late number of the Register an article by Dr. Walker, of New Orleans, giving his method of saving exposed pulps. I agree perfectly with him in regard to the importance of saving pulps alive, but cannot endorse fully the material he recommends for the purpose, viz.: vulcanized rubber. That rubber is a non-conductor and has that virtue in its favor, all will admit; but that it is perfectly harmless for the use Dr. W. recommends it, is a question, which I think would induce all, except rubber worshipers, to look upon it as extremely objectionable, on account of the sulphur and mercury in the compound. Query,—if vulcanized rubber inflames the mouth and gums of persons wearing artificial teeth made of it, which we all know is frequently the case, what effect will it have on as sensitive a part as an exposed pulp, which is more susceptible to any contaminating influences than any other part of the system?

My practice has been for the past five years in nearly every case where the pulp is exposed, to fill over with os-artificiel, being careful not to press the filling too hard on the pulp during its introduction, or it will be liable to produce violent inflammation, and death of the pulp, and likely to result in ulceration. After the material becomes sufficiently hard, I excavate the surplus, leaving just a thin floor in the bottom of the cavity, and complete the operation by filling with gold or tin.

The os-artificiel is one of the best non-conductors we have, and teeth filled in the manner above described, where the pulp is exposed and alive, never trouble the patient in the least when any thing cold or warm comes in contact with them; in fact, I have yet to record the first case where any unpleasantness resulted from this mode of practice, except when first introducing the os-artificiel filling over the pulp, a slight pain is then experienced which seldom lasts longer than from ten to twenty minutes. So far as my experience goes, the pulp seems to contract from the filling.

The idea of filling over an exposed pulp with a plastic material, and the case never troubling the patient, and the palp remaining in its normal condition, may appear incredible to many; but I have operated in this way so long and so successfully, I am convinced that at least one-half of the pulps that are destroyed, might be saved.

POUGHKEEPSIE, N. Y. CHAS. L. HOUGHTON.

